# Gene Editing for Enhanced Swine Production: Current Advances and Prospects

**DOI:** 10.3390/ani15030422

**Published:** 2025-02-03

**Authors:** Won Seok Ju, Seokho Kim, Jae-Yeong Lee, Haesun Lee, Jingu No, Seunghoon Lee, Keonbong Oh

**Affiliations:** Animal Biotechnology Division, National Institute of Animal Science, Rural Development Administration, 1500 Kongjwipatjwi-ro, Iseo-myeon, Wanju-gun 55365, Republic of Korea; jws7895@korea.kr (W.S.J.); jay0i@korea.kr (J.-Y.L.); leehs1498@korea.kr (H.L.); shrkftm@korea.kr (J.N.); sage@korea.kr (S.L.); keonoh@korea.kr (K.O.)

**Keywords:** swine, CRISPR/Cas9 system, gene editing, next-generation sequencing

## Abstract

Traditional pig breeding has improved production traits but faces limitations in genetic diversity, disease resistance, and environmental adaptation. Gene editing technologies, such as CRISPR/Cas9, base editing, and prime editing, enable precise genetic modifications, overcoming these limitations and expanding applications to biomedical research. Here, we reviewed the advancements in gene editing technologies in pigs and explored pathways toward optimized swine genetics for a resilient and adaptive livestock industry. This review synthesizes recent research on gene editing tools applied to pigs, focusing on CRISPR/Cas9 and its derivatives. It examines their impact on critical swine production traits and their role as human disease models. Significant advancements have been made in targeting genes for disease resistance, such as those conferring immunity to porcine reproductive and respiratory syndrome viruses. Additionally, gene-edited pigs are increasingly used as models for human diseases, demonstrating the technology’s broader applications. However, challenges such as off-target effects, ethical concerns, and varying regulatory frameworks remain. Gene editing holds substantial potential for sustainable and productive livestock production by enhancing key traits and supporting biomedical applications. Addressing technical and ethical challenges through integrated approaches will be essential to realize its full potential, ensuring a resilient, ethical, and productive livestock sector for future generations

## Simple Summary

Gene editing technologies are heralding a transformative shift in swine farming. This review explores recent advances in gene editing and its potential to enhance pig production. Beyond traditional breeding methods, cutting-edge technologies, such as CRISPR/Cas9, play a key role in improving traits such as disease resistance, growth rate, and feed efficiency. Gene editing focuses especially on addressing critical challenges, including disease resistance, while also exploring broader future applications. This review not only provides an overview of the current state of gene editing but also examines its practical implications for swine production and the challenges these technologies face moving forward.

## 1. Introduction

Over recent decades, genetic enhancement in pigs has primarily relied on selective breeding, using animals with desirable traits to improve pork production efficiency and quality. While effective, this approach faces limitations when natural genetic variations for specific traits are exhausted. Gene editing technologies address this challenge by introducing novel genetic variations, including traits from other species, or by precisely modifying existing genes. These techniques can enhance or suppress gene expression, delete specific genes, or introduce external genetic sequences.

Earlier gene editing tools, such as mega-nucleases, zinc finger nucleases (ZFNs), and transcription activator-like effector nucleases (TALENs), were effective but technically challenging. The advent of the clustered regulatory interspaced short palindromic repeat (CRISPR)/CRISPR-associated nuclease 9 (Cas9) system revolutionized the field by providing a precise, efficient, and accessible method for targeted genetic modifications. Initially discovered as part of bacterial defenses against viruses, RNA-guided nucleases, such as CRISPR/Cas9 [1,2], have been adapted for genetic engineering due to their ability to create site-specific double-stranded breaks in DNA [3,4]. This process is guided by sequence similarity to genomic regions near protospacer adjacent motifs. Its simplicity and high efficiency have made it a transformative tool in livestock genetic engineering.

In 2014, researchers used CRISPR/Cas9 to create pigs lacking the cluster of differentiation 163 (CD163) receptor [5], rendering them completely resistant to porcine reproductive and respiratory syndromes (PRRS) [6]. This milestone not only shows the potential for disease resistance but also highlights its versatility in enhancing production traits [7], environmental sustainability [8], digestion efficiency [9], and reproductive performance [10]. Additionally, pigs serve as valuable models for improving livestock production traits through somatic cell genome editing, including enhancing disease resistance, growth efficiency, and reproductive performance [11,12]. The growing application of gene editing in the livestock industry underscores its potential to improve animal welfare and production efficiency. This review focuses on these groundbreaking advancements, particularly in the context of swine genetic enhancement.

## 2. Methodological Framework

To ensure rigor and transparency, we adopted a systematic approach based on well-established guidelines for conducting systematic literature reviews, as outlined by Kitchenham (2007) [13]. This framework enhances reproducibility in selecting, evaluating, and synthesizing relevant studies. The following sections outline the key steps of our review process.

### 2.1. Research Objectives and Search Strategy

Our primary objective was to examine advancements in gene editing technologies for swine production, focusing on their applications, challenges, and prospects. To achieve this, we conducted comprehensive searches across multiple academic databases, including PubMed, Web of Science, and Scopus. The search strategy employed a combination of keywords such as “gene editing”, “swine production”, “CRISPR technology”, “next-generation sequencing”, and “livestock”. Boolean operators were applied to ensure broad yet precise coverage of relevant literature.

### 2.2. Inclusion and Exclusion Criteria

To ensure the relevance and quality of selected studies, we established clear inclusion and exclusion criteria:

− Inclusion criteria: Peer-reviewed articles published between 2010 and 2024, specifically focusing on gene editing applications in swine.
− Exclusion criteria: Articles unrelated to swine or gene editing, non-English publications, and studies without full-text access.

### 2.3. Data Analysis and Assessment

Metadata from the selected articles were systematically extracted, including publication year, study design, gene editing tools employed, and primary research outcomes. The data were categorized and analyzed to identify key trends, technological advancements, and practical applications in swine gene editing. Additionally, bibliometric indicators, such as citation trends and journal scope, were assessed to ensure the inclusion of high-impact studies.

## 3. Historical Perspectives on Pig Genetics and Breeding

### 3.1. Traditional Breeding Approaches

Historically, pig breeding has relied extensively on selective breeding, where animals are chosen based on observable phenotypic traits such as growth rate, fertility, and meat quality. This phenotype-driven approach requires keen observational skills and extensive expertise, as decisions are based on physical characteristics and performance records. While traits such as growth and meat quality are relatively straightforward to evaluate, more complex or less observable traits, such as disease resistance or reproductive efficiency, are often overlooked or misestimated [14,15].

The success of selective breeding depends largely on the heritability of target traits and the availability of detailed performance data across generations. For instance, breeders need comprehensive records of offspring performance to accurately determine parental breeding value. However, many economically important traits exhibit low heritability, indicating substantial influence from environmental factors, which complicates consistent genetic improvement [15]. Additionally, the process is time-intensive, often requiring several generations and many years to achieve noticeable progress.

Another major challenge is the risk of inbreeding. To enhance desirable traits, breeders sometimes resort to mating closely related individuals, which reduces genetic diversity [16]. This loss of diversity can increase the prevalence of harmful genetic conditions, weaken immunity, and lower overall reproductive efficiency, threatening the long-term sustainability of breeding programs [17].

### 3.2. Evolution of Genomic Tools in Swine Breeding

The advent of molecular genetics marked a transformative period in pig breeding, drastically improving the speed and precision of genetic gain. A key innovation is genomic selection, which utilizes genome-wide DNA markers to accurately predict an animal’s breeding value [18]. Unlike traditional methods, genomic selection allows breeders to assess traits early in life, even before phenotypic traits are expressed. This reduces the generation interval, allowing faster genetic progress and accelerating the introduction of beneficial traits into pig populations [19,20].

Genomic selection has been instrumental in improving complex traits such as feed efficiency, disease resilience, and reproductive performance—traits that were previously difficult or expensive to measure. By integrating genetic data directly into breeding decisions, breeders can optimize traits such as lean meat percentage and carcass quality while ensuring productivity gains. Additionally, genomic selection facilitates the simultaneous improvement of multiple traits, fostering a balanced approach that considers productivity, animal health, and welfare. Marker-assisted selection complements this by using specific DNA markers linked to desirable traits, making it especially effective for improving traits controlled by a small number of genes [21]. Additionally, the identification and mapping of quantitative trait loci (QTLs) for complex traits have also provided valuable insights into the genetic architecture of traits such as disease resistance and meat quality [22].

The emergence of gene editing technologies, particularly CRISPR-Cas9, has further revolutionized pig breeding [6,23,24,25,26,27]. These tools enable precise modifications of specific genes to enhance traits such as disease resistance and meat quality. However, gene editing also raises ethical and regulatory challenges, including societal concerns about genetic modifications and the need for stringent oversight to prevent unintended consequences [28,29].

### 3.3. Maintaining Genetic Diversity: A Core Challenge

While advancements in genomic tools have accelerated genetic progress in pig populations, maintaining genetic diversity remains equally critical [30]. Rapid genetic gains often involve intense selection, which, if not carefully managed, can narrow the genetic base. A loss of genetic diversity increases the risk of inbreeding, leading to higher frequencies of deleterious alleles, reduced fertility, and increased susceptibility to diseases [30]. To mitigate these risks, breeding programs must balance genetic gain with the conservation of genetic diversity.

Crossbreeding continues to be a vital strategy for maintaining diversity and leveraging heterosis (hybrid vigor), which enhances traits such as growth rate and reproductive efficiency [31,32]. Furthermore, modern genomic tools allow breeders to monitor inbreeding coefficients and assess genetic variability within populations, enabling more informed mating decisions that prioritize diversity conservation [33,34]. Genetic diversity is fundamental to ensuring that pig populations are resilient and capable of adapting to new environmental challenges, disease threats, and market demands, thereby safeguarding the sustainability of genetic improvement efforts.

## 4. Genetic Modification

### 4.1. Germline Modification

Germline modification refers to altering the genetic material of an organism in a way that allows the resulting genetic changes to be passed down to future generations [35,36,37,38,39,40]. For such modifications to be heritable, they must occur within germline cells. The two primary strategies for achieving germline modification are embryonic manipulation and cellular modification techniques, each with distinct advantages and limitations.

#### 4.1.1. Direct Embryonic Manipulation

In a groundbreaking study, Gordon and his co-workers reported the first generation of a transgenic mouse, ushering in a new era in mammalian germline engineering [41]. This approach involves assembling a DNA construct with the desired genes under appropriate regulatory control, microinjecting it into the pronucleus of donor zygotes, and implanting these zygotes into pseudo-pregnant recipient animals [41]. However, outcomes are often stochastic, ranging from no integration to multiple transgene copies, leading to inconsistent expression, mosaicism, and risks of insertional mutagenesis [42]. Therefore, multiple transgenic founders must be screened for proper integration and expression.

In pigs, the application of this method is further complicated by the high lipid content in zygotes, which obscures the pronuclei [43,44], making transgene injection more challenging. Despite these limitations, zygotic injections were the primary genetic manipulation method for two decades and have been used with variable success in pigs [45,46,47]. However, the efficiency of this method was often limited by challenges such as mosaicism, inconsistent expression, and difficulty in achieving precise genetic modifications.

The advent of CRISPR/Cas9 and other programmable editors has addressed many of these issues by enabling more precise, efficient, and targeted gene editing, revolutionizing germline engineering in pigs. Loss-of-function studies, for instance, can create gene-knockout animals by directly microinjecting editors into oocytes [48] or zygotes [49]. CRISPR’s affordability and ease of use (microinjection or electroporation) have democratized germline editing, making it accessible worldwide. Techniques such as i-GONAD, which delivers reagents into the oviduct followed by electroporation, have further simplified germline editing [50].

While zygotic injections have inherent challenges, such as mosaicism and inconsistent expression, advancements in CRISPR/Cas9 technology have significantly improved the precision and efficiency of gene editing in zygotes. One substantial advantage of editing in zygotes, especially with CRISPR/Cas, is the ease of access to the target gene of interest, particularly those located in a heterochromatic state in somatic cells. This is partly due to the unwinding of maternal and paternal chromosomes and the relatively relaxed state of chromatin before pronucleus formation. When zygotes are recovered from in vivo-fertilized zygotes, the efficiency of pregnancy is notably high, with the added benefit of directly introducing the intended genetic modifications into the target genome. Recent advancements in the in vitro culture and fertilization of porcine zygotes, with efficiencies approaching those of in vivo-derived embryos, are expected to expand germline editing efforts [51].

Nevertheless, apart from the ease of delivery, a major limitation of this approach is its inherent mosaicism, with the resulting offspring exhibiting distinct somatic and germline modifications. This complicates the screening of heritable mutations in large animal models, such as pigs. Mosaicism is particularly pronounced in methods involving homology-directed repair (HDR)-based targeting [52]. Various strategies have been proposed or assessed, including adjusting reagent concentrations, selecting optimal reagents, modifying targeting oligonucleotides/plasmids, altering injection timings, and employing CRISPR inhibitors. However, each allele and trait necessitate independent validation, and thorough in vitro validation assays should be conducted before committing to embryo transfers, the associated protracted gestation period, or reaching puberty.

#### 4.1.2. Somatic Cell Nuclear Transfer

Nuclear transfer dates to the era of zygotic injections. The theoretical basis for somatic cell nuclear transfer (SCNT) was first proposed by Spemann in 1938 [53]. In 1952, Briggs and King were the first to experimentally demonstrate nuclear cloning by transplanting a nucleus from a blastula-stage frog embryo into the cytoplasm of an enucleated frog egg [54]. In 1981, Illmensee reported the creation of clonal mice using SCNT; however, this claim was later questioned [55]. Fifteen years after the generation of the first transgenic mouse and nearly three decades after amphibian cloning, Campbell et al. successfully cloned a sheep named Dolly [56]. This success was soon extended to pigs [57,58]. The SCNT process involves several critical steps, each of which may substantially affect cloning efficiency. These steps include genetic alteration of donor cells, removal of metaphase chromosomes from metaphase II-arrested oocytes (enucleation) via aspiration or bisection, introduction of donor cell nuclei, either through fusion with the enucleated oocyte by an electrical pulse or direct injection into the cytoplasm, activation of the reconstructed embryos, and eventual culture and transfer of embryos into synchronized surrogate recipients [59,60]. Due to the relatively long gestation periods and time to reproductive maturity of pigs, breeding hemizygous founders to achieve homozygosity presents a significant challenge. To achieve homozygosity, a two-step approach is typically employed (1 in 10^6^–10^7^ in cultured cells): first, a round of embryo transfer is performed to harvest fetal fibroblasts with hemizygous modifications; then, a second round of gene targeting is conducted in these fibroblasts to introduce biallelic modifications before processing with SCNT. This ensures that the resulting offspring will carry the desired homozygous genotype [61,62,63]. However, achieving homozygosity remains a cost-prohibitive challenge because of the time and resources involved in these steps.

At the cellular level, the somatic cells commonly used in SCNT are fetal fibroblasts. These cells proliferate slowly and have a limited lifespan in culture, which can make the process challenging. Additionally, gene modification efforts in these cells often result in hemizygosity [61,63]. Due to the long gestation period and the extended time required for pigs to reach reproductive maturity, breeding hemizygous founders to achieve homozygosity is both time-consuming and cost-prohibitive.

A round of embryo transfer is often conducted to harvest fetal fibroblasts with hemizygous modifications, followed by a second round of gene targeting and SCNT to generate offspring with biallelic modifications. To overcome this, one approach involves conducting an initial round of embryo transfer to obtain fetal fibroblasts with hemizygous genetic modifications. This is then followed by a second round of gene targeting and SCNT to produce offspring with biallelic (homozygous) modifications. However, the efficiency of SCNT remains low, and the process is technically demanding. Incomplete embryo reprogramming further complicates the procedure, often resulting in abnormalities in cloned animals, such as lameness, respiratory defects, immunodeficiency, obesity, and premature death [64,65]. These issues persist despite significant advancements in SCNT technology.

While gene-targeting efficiency in somatic cells is generally low, introducing double-stranded breaks at the target site can significantly enhance the success rate of gene modification [66]. This improved efficiency allows for complex genetic modifications, such as achieving homozygosity, to be completed in a single round of in vitro gene targeting. When pre-screened clonal cell lines are utilized for SCNT, the resulting litter of cloned animals uniformly carries the desired genetic modification. However, this strategy does not address the inherent risks of incomplete embryo reprogramming. Despite these challenges, SCNT remains the most widely used method for germline genetic engineering in pigs due to the lack of viable alternative technologies.

Embryo-based germline editing is typically preferred in agriculture, where such modifications are performed on elite founder animals within nuclear herds, enabling their incorporation into breeding programs [67]. Cell-based approaches are more frequently utilized when genetic modifications must be introduced into rare or less prolific breeds, such as Yucatan, Ossabaw, and other miniature pigs, or when phenotypic outcomes of loss-of-function or overexpression studies must be assessed before deployment in a commercial environment [68,69]. Despite the inherent trade-offs of each method, the advent of genome editors has substantially accelerated the adoption of germline engineering to address critical agricultural priorities [67,68,69]. The scope of genetic modifications is limited by imagination and biological constraints. The genetic modification that is conceivable and compatible with biological systems will likely be realized. Advances in the functionality and applicability of genome editing tools are elaborated on in the following section.

### 4.2. Genome Editing Techniques

**Figure 1:**
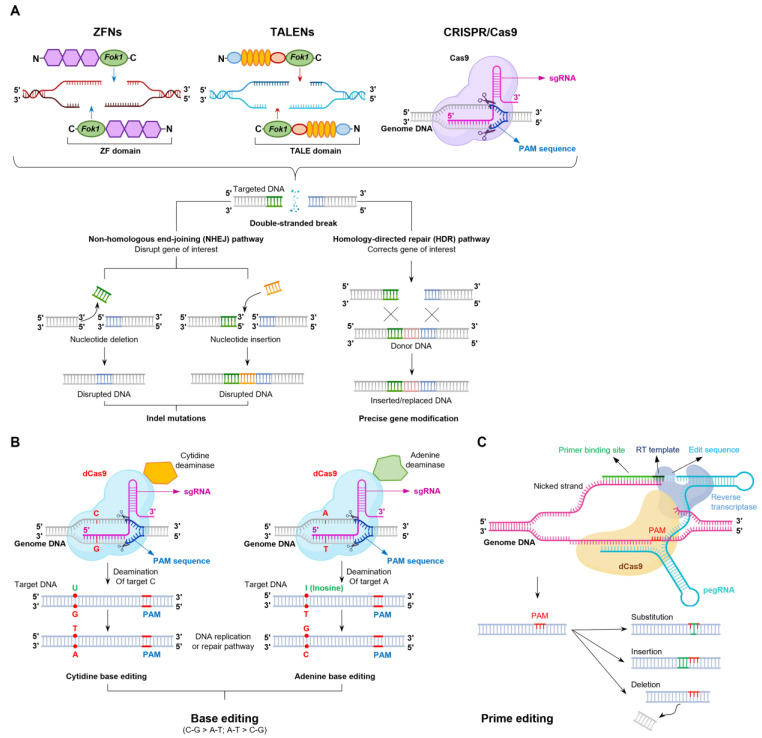
Applications of the genome editing technologies. (**A**) Mechanisms of ZFNs, TALENs, and CRISPR/Cas9. ZFNs, TALENs, and CRISPR/Cas9 technologies enable accurate and efficient genetic modification by generating site-specific DNA double-stranded breaks, which are resolved through NHEJ and HDR repair mechanisms. NHEJ-driven repair introduces indels of varying sizes, while HDR-based repair, with the presence of donor DNA, facilitates precise point mutations and gene replacements. The sgRNA consists of two components: a guide RNA complementary to the target gene and a tracrRNA, which associates with the Cas9 enzyme. The sgRNA guides Cas9 to a specific genomic location adjacent to a PAM sequence, introducing double-stranded breaks 3-4 bp upstream of the PAM. (**B**) Mechanisms of base editing technology. The cytosine base editing, which combines dCas9 with cytidine deaminase, facilitates the conversion of the C-G pair to A-T; the adenine base editing, incorporating dCas9 and adenine deaminase, enables the conversion of the A-T pair to C-G. (**C**) Prime editing technology. The prime editing technique is a modified fusion protein consisting of nCas9 and RT. The nCas9 generates a single-stranded break on the non-target DNA strand. The exposed 3′ end then pairs with the 3′ end of the pegRNA, and the RT domain catalyzes the reverse transcription process. This results in the incorporation of the desired sequence changes, as specified by the pegRNA, into the newly synthesized DNA strand. The edited 3′ flap and unmodified 5′ flap undergo equilibration, followed by 5′ flap cleavage and ligation, ultimately leading to the stable integration of the modification into the genome. Abbreviation: A, adenine; bp, base pair; C, cytosine; CRISPR/Cas9, clustered regularly interspaced short palindromic repeats (CRISPR)/CRISPR-associated protein 9 (Cas9); dCas9, Cas9 endonuclease dead or catalytically inactive Cas9; G, guanine; HDR, homology-directed repair; I, inosine; Indel, insertion-deletion; nCas9, nickase Cas9; NHEJ, non-homologous end-joining; PAM, protospacer adjacent motif; pegRNA, prime editing-guide RNA; RT, reverse transcriptase; sgRNA, single-guide RNA; T, thymine; TALEN, transcription activator-like effector nuclease; U, uracil; ZFN, zinc finger nuclease.

The advancement and implementation of gene editing technologies in swine production have considerably enhanced the efficiency of producing genetically modified pigs. Over the last 15–20 years, various genome editing tools have emerged. These technologies are fundamentally based on nucleases that target specific sites within the genome, resulting in DNA cleavage and the formation of double-strand breaks (DSBs). Subsequently, DSBs are repaired via the cell’s inherent mechanisms. The most employed and efficient repair pathway is non-homologous end-joining [66,70,71], which is error-prone and often leads to small insertions or deletions (indels) [72,73]. These indels can vary in size and may induce frameshift mutations in the coding sequence or delete translation start sites, ultimately resulting in gene knockouts [66,72,73]. When multiple guide RNAs are intentionally designed to target distinct sites within a gene, the CRISPR/Cas9 system can facilitate the indels of intervening sequences, thereby leading to targeted gene knockouts [74]. Alternatively, when donor templates or oligonucleotides are available, the HDR pathway can be employed to introduce precise modifications from the donor template at the nucleotide level [75,76,77]. This facilitates the insertion of transgenes, exon swapping, or even single-nucleotide alterations into target cells [5,78]. However, the efficiency of achieving the desired modifications in cells or embryos through HDR is often low, typically ranging from 1% to 10%, depending on factors such as the cell type, delivery method, and the availability of a suitable donor template [75,76,77]. Figure 1 illustrates the overview of the mechanisms and advancements of ZFNs, TALENs, CRISPR/Cas9, and next-generation editing technologies, including base and prime editing techniques.

#### 4.2.1. ZFNs

Early-generation tools, such as ZFNs and TALENs, have been instrumental in establishing methods for targeted genetic modifications. Both tools use engineered proteins to create DSBs at specific DNA sequences, which are subsequently repaired by the cell’s natural mechanisms, often resulting in gene knockout and insertion. ZFNs were among the pioneering technologies enabling precise targeting of virtually any DNA sequence, making them one of the first tools for genome editing in mammalian cells [79]. Zinc fingers are protein domains that bind specific DNA sequences, typically recognizing unique three-nucleotide sequences. These zinc fingers are arranged in a series of 3–15 repeats, with the number of binding motifs influencing sequence recognition specificity [80]. This DNA-binding specificity is coupled with the nuclease domain *Fok1,* which cleaves DNA [81,82]. Although ZFNs were used for efficient modifications in somatic cells, their application involves inherent challenges [83]. These include complex assembly processes where ZF domains in tandem can influence each other’s specificity, making the design and implementation of ZFNs intricate and less straightforward [83]. A notable example is the generation of enhanced green fluorescent protein knockout pigs, which fully utilize ZFN technology [84]. Since then, ZFNs have been widely applied to produce knockout pig models with specific genetic modifications [85,86].

#### 4.2.2. TALENs

The next technology, TALEN, faced an unfortunate fate, overshadowed by the capabilities of ZFNs and the CRISPR/Cas9 system. Structurally, TALENs and ZFNs share the *Fok1* gene-cleavage domain but differ in their gene recognition domains. ZFNs use zinc finger motifs [87], while TALENs rely on TALE proteins, which are structurally distinct from zinc finger proteins. However, they use the same restriction enzyme *Fok1* to cut the target gene [88,89,90,91,92].

Similar to the creation of an enhanced green fluorescent protein knockout model using ZFNs [84], Carlson et al. successfully applied TALEN technology to modify the low-density lipoprotein receptor (*LDLR*) gene in pigs, though production was not pursued [93]. Since the generation of immune-deficient pigs [94,95], no further reports of TALEN use in pigs have emerged. While TALENs succeeded ZFNs and showed promise in the scientific community, they were soon eclipsed by the advent of the simpler and more advanced CRISPR/Cas9 technology. Despite their precision, TALENs are labor-intensive, time-consuming, and require complex protein engineering, which has limited their widespread adoption.

#### 4.2.3. CRISPR Technology

With the emergence of third-generation gene editing technology CRISPR/Cas9, genome editing has entered a revolutionary era marked by considerably higher accuracy and efficiency compared with earlier methods. The CRISPR/Cas9 system, derived from a bacterial immune defense mechanism [96,97], uses Cas proteins to create DSBs in the DNA of invading bacteriophages, leading to the destruction of their genetic material [1,2]. This process relies on sequence alignment at genomic loci adjacent to protospacer adjacent motifs.

The system employs a guide RNA (gRNA) to direct the Cas9 enzyme to a specific DNA sequence [98], where it precisely cuts the target. Cellular repair mechanisms are then activated [1,3,99,100,101,102], allowing researchers to delete or insert genes with remarkable accuracy [3,4]. For example, in 2014, Whitworth and her co-workers used CRISPR/Cas9 to generate pigs lacking CD163 receptors [5], rendering them completely resistant to porcine reproductive and respiratory syndrome virus (PRRSV) [6]. The expression of the Cas9 protein associated with an appropriate gRNA allows in vivo gene editing, providing a more versatile and precise method for studying target gene functions. For instance, cells derived from Cas9-expressing pigs demonstrated successful deletion of the *CD163* gene upon the introduction of a CD163-specific gRNA [103].

Beyond developing disease-resistant animals, CRISPR/Cas9 has been widely applied to enhance production traits [7], minimize environmental impacts [8], improve digestion efficiency [9], and advance reproductive capabilities [10]. Its ease of use, versatility, and ability to simultaneously target multiple genes have established CRISPR/Cas9 as the dominant tool in various gene editing applications.

#### 4.2.4. Base Editing and Prime Editing

Although the CRISPR/Cas9 system has revolutionized gene editing, it faces notable limitations in inserting specific DNA sequences into a genome. Using the natural HDR mechanism for precise DNA template insertion is inefficient, often leading to insertion errors and high rates from 10% to 50% of indels [72]. To address these challenges, advancements in gene editing have introduced modified CRISPR/Cas9 components that enable point mutations in DNA or RNA without causing DSBs [104,105,106]. These innovations include two primary types of base editors: cytosine- and adenine-based.

The first-generation base editor (BE1) combines catalytically inactive Cas9 (dCas9) with a cytosine deaminase enzyme, converting cytosine to uracil. During DNA replication, uracil is recognized as thymine, leading to a C-to-T or G-to-A substitution, depending on the target DNA strand [107]. The second-generation base editor (BE2) was improved upon BE1 by adding a uracil DNA glycosylase inhibitor to enhance the conversion of U.G pairs to T.G pairs [108]. The third-generation base editor (BE3) introduced Cas9 nickase (nCas9) in place of dCas9, which “nicks” DNA and takes advantage of the cellular mismatch repair system to drive the desired edit [109]. A fourth-generation base editor (BE4) co-expresses BE3 with a uracil DNA glycosylase inhibitor, enhancing C-to-T conversion efficiency [106]. While base editing efficiently generates four types of transition mutations (C-to-T, G-to-A, A-to-G, and T-to-C), it cannot create transversion mutations [107]. Ongoing research aims to expand the capabilities of base editors, including addressing the challenge of accurately targeting single bases when multiple similar bases are present in a sequence or located near the gRNA binding site.

To overcome base editing’s limitations, prime editing was developed. This technique enables insertions, deletions, and all 12 possible base-to-base conversions without requiring DSBs or donor DNA templates [110]. It minimizes off-target effects and enhances genome-editing precision [110]. Prime editing employs Cas9 nickase (nCas9) fused to a reverse transcriptase (RT-nCas9), which is transfected into cells along with a prime editing guide RNA (pegRNA). Unlike the traditional CRISPR/Cas9, the pegRNA not only guides the enzyme to the target sequence of DNA, similar to single guide RNA, but also carries the new sequence for replacement [110]. After target recognition, the strand containing the PAM is nicked, allowing the pegRNA to bind [111]. A 3’OH DNA flap is created, which acts as a primer for the reverse transcriptase to copy the new sequence encoded by the pegRNA [112]. Prime editing has been successful in human HEK293T cells (achieving editing efficiencies between 20% and 50%) [110], mouse cells (8–40%), and zygotes (44–75%) [113]. However, its application to genetically modified livestock remains unexplored.

These advancements have enabled precise trait modifications, such as enhanced disease resistance, improved meat quality, and reduced environmental impacts, by lowering methane emissions from livestock. As technology continues to evolve, the potential for widespread, cost-effective, and rapid gene editing in pigs has become increasingly feasible.

### 4.3. Challenges and Limitations

Gene editing in swine production has transformative potential for improving key traits, such as disease resistance, growth efficiency, and reproduction. However, several technical and conceptual challenges must be addressed before their widespread application in commercial settings.

#### 4.3.1. Technical Limitations

One of the foremost challenges is the precision and efficiency of gene editing technologies. Although tools such as CRISPR/Cas9, TALENs, and ZFNs have significantly advanced this field, the risk of off-target effects remains a persistent concern [114]. Unintended editing can result in deleterious mutations, potentially compromising animal health or productivity [115]. Although advancements in more sophisticated editing techniques, such as base and prime editing, have shown promise in reducing off-target activity, their reliability in large-scale applications remains an ongoing research area [110]. Additionally, efficient germline editing poses technical challenges. Methods involving embryonic manipulation, such as embryonic stem cell-based modifications and SCNT, require highly specialized expertise and cutting-edge infrastructure [116]. Post-manipulation survival rates of embryos remain inconsistent, and achieving viable gene-edited animals with consistent phenotypic outcomes remains challenging [117].

#### 4.3.2. Biological and Physiological Constraints

Biological variability also presents a significant limitation to the efficacy of gene editing. The outcomes of editing interventions can differ widely depending on genetic background, complicating the development of universally applicable gene edits [83]. Gene knockouts intended to confer disease resistance may vary in effectiveness across pig breeds and farm environments, complicating broader applications. Mosaicism, a phenomenon wherein not all cells carry the desired genetic alterations, is another complicating factor, particularly in germline editing [118]. This variation in cellular modification introduces challenges in achieving uniform gene editing across tissues and complicates breeding strategies to perpetuate edited traits across generations.

#### 4.3.3. Ethical and Regulatory Considerations

Ethical concerns regarding gene editing in livestock are critical issues that require careful consideration. There are social implications of modifying animal genomes, particularly for agricultural purposes, raising questions regarding animal welfare, food safety, and potential environmental impacts [119]. While genetically editing livestock offers notable benefits, public skepticism regarding GMOs and genetically modified animals poses a major barrier to acceptance [120]. Addressing these concerns requires transparency, rigorous safety assessments, and public engagement to build trust in the technology. The regulatory landscape of gene editing in animals adds another layer of complexity. Regulatory frameworks differ substantially among regions, with some adopting permissive policies and others imposing stringent restrictions or outright bans [121]. This regulatory heterogeneity complicates the commercialization and global trade of genetically edited pigs, necessitating efforts to harmonize international standards and streamline the approval process. A balanced regulatory approach that ensures both safety and innovation is imperative to fully realize gene editing’s potential.

#### 4.3.4. Economic and Logistic Challenges

From an economic perspective, implementing gene editing technologies in swine production presents significant financial and logistical challenges. Editing, selecting, and breeding animals with desired traits are resource-intensive, demanding advanced technologies, expertise, and substantial financial investments [122]. These barriers are particularly pronounced for small-scale producers who may lack the capital and technical expertise to effectively adopt these technologies. Moreover, the current cost-benefit ratio for genetically edited livestock remains uncertain. Fan et al. (2024) highlighted additional considerations, including the role of market dynamics and logical constraints, in shaping the economic feasibility of gene-edited livestock [123]. Given the fluctuating market conditions and ongoing consumer demand for traditionally bred or non-GMO livestock products, the long-term economic viability of gene-edited animals in commercial production is yet to be fully established. For many producers, the high upfront costs associated with technology may outweigh the perceived advantages, particularly in the absence of clear market incentives.

## 5. Next-Generation Sequencing and Its Applications

Advancements in sequencing technologies have fundamentally reshaped our understanding of swine genetics, driving advancements in breeding methodologies and disease control strategies with unprecedented precision. Each generation of sequencing platforms has introduced new technical capabilities that have transformed genomic research.

The evolution of sequencing technologies has been presented below, with an overview of first to fourth-generation platforms.

### 5.1. First-Generation Sequencing

First-generation sequencing, particularly Sanger sequencing and Maxam-Gilbert sequencing, represents a marked advancement in DNA sequencing technologies. These technologies represent significant milestones in the history of DNA sequencing, establishing the groundwork for subsequent advancements. First, Maxam-Gilbert sequencing, developed by Allan Maxam and Walter Gilbert in 1977, was the first method to directly sequence DNA [124]. This chemical-based approach uses radiolabeled DNA and selective cleavage at specific nucleotide bases, generating fragments that are separated by gel electrophoresis. Despite its historical significance, Maxam-Gilbert sequencing is rarely used today due to its reliance on hazardous chemicals, labor-intensive protocols, and limited scalability [124]. Next, introduced by Frederick Sanger in 1977, this method uses dideoxynucleotides to terminate chains during DNA synthesis, creating DNA fragments of varying lengths that can be separated using gel electrophoresis [22]. Although labor-intensive and low throughput, Sanger sequencing has been instrumental in early genomic research and served as the foundational standard for DNA sequencing for several decades. It played a crucial role in the initial mapping of critical genetic traits in swine production, particularly identifying QTLs linked to growth, meat quality, and disease resistance [22].

### 5.2. Second-Generation Sequencing

Second-generation sequencing (SGS), commonly referred to as next-generation sequencing (NGS), has enabled large-scale whole-genome sequencing (WGS) and marked a significant leap forward in sequencing technology, characterized by improved throughput and scalability. Technologies such as Illumina sequencing-by-synthesis and Roche 454 pyrosequencing generate millions of short DNA reads in parallel, considerably reducing the time and cost associated with sequencing [125]. The sequencing of the pig reference genome (Sus scrofa 10.2) using Illumina technology [126] helped identify genetic markers linked to key production traits, including feed efficiency, reproduction, and disease resistance. NGS has also enabled genome-wide association studies (GWAS) to uncover loci associated with economically important traits and detect copy number variations (CNVs), enhancing our understanding of genetic diversity and its connection to traits such as immune response and feed efficiency [127]. Its parallel sequencing capability allows for rapid resequencing of entire populations, aiding in identifying rare variants with substantial phenotypic value.

### 5.3. Third-Generations Sequencing

Third-generation sequencing (TGS), including Pacific Bioscience’s single-molecule real-time sequencing and Oxford Nanopore’s nanopore sequencing, generates much longer read lengths without amplification, thereby mitigating sequencing biases and facilitating the detection of epigenetic modifications such as DNA methylation [128]. In swine production, TGS has revealed structural variants associated with traits such as growth, fat deposition, and meat quality that were previously undetectable using short-read technologies [129]. Long reads from TGS also enable more precise resolution of repetitive elements and gene locations, offering critical insights into genomic regions that are often difficult to map with shorter reads. Furthermore, the ability to detect full-length transcript isoforms provides a more accurate view of gene expression, enhancing understanding of alternative splicing events that may play a role in complex traits [130].

### 5.4. Fourth-Generation Sequencing

The latest advancements in sequencing technologies include fourth-generation sequencing, single-cell sequencing, and spatial transcriptomics. These technologies offer unprecedented resolution, allowing researchers to investigate gene expression at the single-cell level, offering insights into cellular heterogeneity and tissue-specific functions [131]. In swine production, single-cell RNA sequencing has demonstrated the potential for profiling immune cell populations, elucidating disease resistance mechanisms, and enhancing precision breeding by linking cellular phenotypes with genetic markers [132]. Additionally, spatial transcriptomics has enabled researchers to visualize gene expression in specific tissue sections, providing crucial insights into traits such as muscle development and fat deposition [133]. These approaches allow a detailed understanding of the spatial architecture of tissues, which is critical for evaluating how localized gene expression contributes to economically important traits. Table 1 provides a comprehensive summary of the key features, limitations, and applications of sequencing technologies described above.

## 6. Key Traits and Target Genes for Pig Genetic Improvement

### 6.1. Growth Rates

Targeting growth-regulatory genes such as myostatin (MSTN) is highly effective in improving growth traits. MSTN serves as a negative regulator of muscle growth, and its inactivation through CRISPR/Cas9 gene editing leads to significant muscle hypertrophy and leaner carcasses [134]. MSTN editing enhances muscle mass, reduces intramuscular fat [135], and improves feed efficiency [136], benefiting production systems. However, studies indicate the impact of MSTN inactivation on other physiological pathways, including energy metabolism and insulin sensitivity, requiring careful monitoring to avoid adverse effects on animal welfare [137,138]. Moreover, MSTN knockout has implications for not only growth traits but also metabolic health and resilience to environmental stressors. Recent research suggests that MSTN-edited pigs exhibit altered energy expenditure and improved resilience to temperature fluctuations [139]. These findings are crucial for adapting swine production to diverse environmental conditions, particularly in regions prone to climatic variability.

### 6.2. Modification in Carcass Composition and Enhancements in Meat Quality

Advancements in meat quality have been made through the expression of a fatty acid desaturase gene derived from spinach, aimed at boosting linoleic acid levels in swine. Unlike other species, mammals are unable to synthesize omega-6 and omega-3 fatty acids owing to the absence of the necessary desaturases. Consequently, the incorporation of the Delta12 fatty acid desaturase gene from spinach in pigs enables the efficient production of these essential fatty acids, leading to elevated linoleic acid concentrations [140]. Furthermore, a team at the University of Missouri developed pigs capable of producing their own omega-3 fatty acids, typically sourced from fish oils and beneficial to human health. This was achieved by expressing a humanized version of the *Caenorhabditis elegans* gene, hfat1, which resulted in an enhanced omega-3 fatty acid profile in comparison to wild controls [141].

### 6.3. Enhancement of Thermoregulatory Efficiency in Pigs

Newborn piglets exhibit poor thermoregulatory ability owing to an inactive uncoupling protein 1 (*UCP1*) gene, which plays a crucial role in regulating body temperature and fat storage. A genetic event occurring approximately 20 million years ago led to the deletion of exon 3 through 5 of the *UCP1* gene in pigs [142]. In modern pig farming, sows are often housed in farrowing crates equipped with heating elements, resulting in increased energy use and production expenses. To restore proper thermoregulation, researchers employed CRISPR/Cas9 technology to introduce the mouse adiponectin-*UCP1* gene into the native *UCP1* locus of pigs [143]. The genetically modified pigs displayed enhanced thermoregulatory responses to cold stress, with reduced fat accumulation, all while maintaining normal physical activity and energy consumption levels [143].

### 6.4. Reproduction and Fertility

Reproductive performance, particularly litter size, is critical for the economic sustainability of pig production. Genetic improvements have been facilitated by identifying genetic markers linked to ovulation rate, uterine capacity, and embryonic survival. Key genes such as Estrogen Receptor 1, Follicle-Stimulating Hormone Beta, and RBP4, have been recognized as critical contributors to reproductive success [144,145,146,147]. Additionally, a previous study identified a significant association between an SNP in ZP3 and male fertility in Duroc pigs, demonstrating a correlation with the total number of piglets born [148]. Multiple studies suggest ZP3 could be a potential target for improving fertility and reducing pre-implantation embryo loss in pigs [149,150]. Furthermore, CatSper subunits in boar spermatozoa regulate motility during capacitation, with bicarbonate exposure increasing Ca^2+^ channel mRNA expression and affecting motility, similar to CatSper inhibition, suggesting their essential role in fertility [151,152].

### 6.5. Disease Resistance

Porcine has significant value as a resource in the agricultural and livestock industries. Considerable economic losses caused by various diseases such as African swine fever (ASF), classical swine fever virus (CSFV), Nipah virus disease, Japanese encephalitis virus, PRRSV, transmissible gastroenteritis virus (TGEV), and porcine delta coronavirus (PDCoV) have affected the porcine industry in the world [153,154,155,156,157,158,159]. Traditional approaches for developing porcine antiviral vaccines are time- and labor-intensive [160]. Currently, the CRISPR/Cas9 system precisely targets and excises exogenous DNA originating from viruses or plasmids [2]. By leveraging biotechnology, researchers can develop groundbreaking tools to protect domestic pigs against pathogenic bacterial and viral infections.

#### 6.5.1. PRRSV

The CD163 is expressed on porcine monocytes and macrophages and serves as a crucial receptor for PRRSV infections [161,162]. Using CRISPR/Cas9-edited somatic cells, *CD163* knockout pigs were generated through the SCNT technique, which demonstrated resistance to PRRSV and proved resistant to PRRSV [5,6,163]. To preserve CD163’s biological functions, researchers specifically targeted the scavenger receptor cysteine-rich domain 5 (SRCR5) encoded by exon 7 of *CD163* [163]. They either disrupted SRCR5 or substituted exon 7 of porcine *CD163* with the corresponding exon of human *CD163*-like1 (*hCD163L1*) [164]. The deletion of CD163 SRCR5 conferred in vivo resistance to PRRSV-1 and in vitro resistance to both PRRSV-1 and PRRSV-2 while maintaining the biological function of CD163 [165]. Similarly, replacing the porcine CD163 SRCR5 domain with a human *CD163*-like SRCR8 domain rendered resistance to PRRSV-1 but not PRRSV-2 [166]. Recently, eliminating a 41-amino acid fragment containing the ligand-binding pocket of the SRCR5 domain in *CD163* provided pigs with complete resistance to PRRSV-2 infection [167].

Although CD163 is the proposed receptor for both PRRSV-1 and PRRSV-2, these viruses exploit different sites on CD163 to establish infection. Thus, partial depletion of CD163 was guided by the analysis of the confirmed sites for PRRSV-1 and PRRSV-2 infections. Researchers are eager to generate pigs that exhibit resistance to multiple viral pathogens. In 2018, Oh et al. devised a multi-resistance approach against foot and mouth disease (FMD) and PRRSV using the CRISPR/Cas9-meditated deletion of the *CD163* gene and incorporating small hairpin RNA (shRNA) into pig fibroblasts [168]. The integrated shRNA targeted the three-dimensional gene of the FMD virus and the open reading frame 7 (*ORF7*) gene of PRRSV. However, the mass production of double-resistant pigs against FMD and PRRSV requires further validation through in vivo viral challenge tests.

Recently, *CD163* and aminopeptidase N (*APN*) double-knockout (DKO) pigs confer complete resistance to PRRSV-2 and TGEV. Notably, an additional viral challenge revealed that DKO pigs showed reduced susceptibility to PDCoV, providing unprecedented in vivo evidence that porcine APN is a receptor for PDCoV [169]. Therefore, multiple key genes could serve as targets for generating pigs resistant to multiple diseases. However, the limited understanding of receptor interactions in many viral diseases may pose challenges to this multi-resistance strategy.

#### 6.5.2. ASFV

Unlike CSFV, the ASF virus (ASFV) is an acute and fatal viral pathogen that affects both domestic and wild pigs. However, the search for an effective vaccine candidate to combat ASFV infections remains unresolved. Researchers initially suggested that ASFV targets CD163+ monocyte subpopulations, but not CD163-ones [170]. Blocking membrane receptors with specific antibodies inhibited ASFV infection, indicating CD163 involvement [170]. However, subsequent studies have disproved CD163 as an ASFV receptor [171]. Further investigations found several factors, such as the epidermal growth factor receptor (EGFR), phosphoinositide 3-kinase (PI3Ks), p21-activated kinase-1 (PAK1), Niemann-Pick C1 (NPC1), and NPC2, as potential factors in ASFV entry, highlighting the complexity of ASFV infection mechanisms [172,173,174].

In 2018, HüBner et al. deployed the CRISPR/Cas9 system to target the double-stranded DNA (dsDNA) genome of ASFV [175]. In vitro trials demonstrated that CRISPR/Cas9 could effectively control ASFV infection. This approach has shown potential for application in indigenous animal hosts, offering direction for ASF prevention strategies [175]. The ASFV *p30* gene presents a viable strategy against ASFV infection, with potential applicability in indigenous animal hosts [175].

#### 6.5.3. Coronavirus

CRISPR/Cas9 plays a pivotal role in the generation of pigs resistant to coronaviruses (CoVs). CoVs are ubiquitous single-stranded RNA (ssRNA) viruses, including TGEV, PEDV, and PDCoV, that cause significant losses in the swine industry [176]. These viruses cause extensive mortality in suckling piglets due to malabsorptive diarrhea and dehydration [177]. The CRISPR/Cas9 system confirmed the essential role of the APN protein, situated on the surface of the intestinal villi, as a key receptor facilitating TGEV infection in swine [178,179]. While TGEV and PEFV primarily infect suckling piglets rather than post-weaning pigs, neonatal piglets lacking APN demonstrate resistance to TGEV infection. However, APN deficiency did not confer immunity against PEDV infection [180]. Additionally, pigs lacking the *CMAH* gene deletion did not show protection against PEDV infection [181]. Consequently, identifying distinct receptors for PEDV is crucial.

#### 6.5.4. CSFV

CSFV is the etiological agent responsible for CSF disease, which induces immunosuppression and renders domestic and wild boars susceptible to secondary opportunistic infections of the gastrointestinal and respiratory systems [182]. Transgenic pigs resistant to CSFV were generated using the CRISPR/Cas9 system combined with RNA interference. Through a CRISPR/Cas9 knock-in approach, small hairpin antiviral RNA (shRNA) was inserted into the swine Rosa26 locus to degrade CSFV RNA. Viral challenge experiments in these pigs demonstrated a significant decrease in CSFV replication, clinical symptoms, mortality rates, and the successful transmission of CSFV resistance to first-generation piglets [183]. Further systematic investigations are warranted to ascertain whether CSFV encodes viral suppressors of RNAi (VSRs) and whether these VSRs can counteract the antiviral RNAi trait in transgenic pigs.

Radical S-adenosyl methionine domain-containing protein 2 (RSAD2) is a cellular protein that exhibits broad-spectrum antiviral activity against DNA and RNA viruses [184,185,186]. In contrast, RNA-dependent adenosine deaminase 2 is a different enzyme involved in RNA editing, playing a role in immune responses in swine and contributing to their resistance to infections. Owing to its antiviral properties, RSAD2 is considered a potential candidate for CRISPR/Cas9 knock-in to develop virus-resistant transgenic pigs. Targeted insertion of the porcine *RSAD2* gene into the porcine Rosa26 locus yielded transgenic swine displaying reduced CSDV and pseudorabies virus (PRV) infections upon viral challenge [187].

### 6.6. Other Potential Disease-Resistant Pig Models

Additional genetically engineered models have been developed to examine disease resilience and receptor-binding capabilities. A notable example is the targeted ablation of transmembrane protease, serine S1, member 2 (*TMPRSS2*) [188]. This model was developed to elucidate the involvement of TMPRSS2 protease in the pathogenesis of swine influenza. Host proteolytic cleavage of influenza hemagglutinin by endogenous proteases is essential for viral infectivity [189,190]. The ablation of this protease may hinder influenza infectivity; however, conclusive data are yet to be published, leaving the model’s efficacy speculative.

Significant progress has been made in attenuating the virulence of CSFV and PRV, two major pathogens causing substantial economic losses. Using the CRISPR/Cas9 technology, porcine models were developed with locus-specific integration of the *RSAD2* gene. In vitro and in vivo infection experiments have demonstrated that overexpression of porcine *RSAD2* in both porcine cell lines and whole animals considerably reduces CSFV and PRV infectivity [187].

Furthermore, gene editing methodologies have been used to create porcine lines that produce human lysozyme (hLZ) in milk, a naturally occurring antimicrobial agent. The consumption of hLZ-rich milk by piglets has been associated with accelerated recuperation from bacterially induced diarrheal diseases [191,192]. Expanding our insights into viral entry mechanisms will facilitate the strategic engineering of pigs with enhanced resistance to diverse pathogens. In an ambitious paradigm of multiplex gene editing, we generated pigs carrying concurrent edits in *CD163*, *SIGLEC1*, and *ANPEP*, thereby endowing the animals with compounded resistance to multiple infections.

## 7. Key Findings from the Systematic Review

Following the systematic screening process, 215 relevant articles were initially identified, of which 82 studies met the inclusion criteria. These studies were further analyzed to highlight the latest advancements in gene editing technologies and their applications in swine production.

### 7.1. Technology Trends and Applications

Our review revealed that CRISPR/Cas9 technology remains the dominant gene editing tool, accounting for 65% of the studies due to its simplicity, efficiency, and versatility. Emerging techniques, such as base editing and prime editing, were identified in 15% of the selected studies, offering the potential for more precise and less error-prone genetic modifications. Several studies have reported significant progress in developing disease-resistant pig models, particularly for the Porcine Reproductive and Respiratory Syndrome Virus (PRRSV) and African Swine Fever Virus (ASFV). Notably, CD163 and APN gene knockout models were widely studied for conferring resistance to these diseases. Additionally, gene editing efforts targeting MSTN and reproductive markers demonstrated promising applications for enhancing productivity, muscle growth, and fertility in commercial swine breeding.

### 7.2. Challenges and Future Directions

Despite rapid progress, several challenges remain. Off-target effects, ethical concerns, and regulatory barriers were recurrent themes in the reviewed literature, highlighting the need for improved gene editing precision and harmonized global regulations. Many studies suggested integrating NGS with advanced genomic prediction models could mitigate current limitations and enhance the accuracy of gene editing applications.

Future research should focus on refining genome-editing tools, improving long-term safety assessments, and addressing ethical considerations to facilitate broader adoption in commercial livestock production.

## 8. Conclusions

The advent of gene editing technologies represents a monumental advancement in swine production, offering unprecedented potential to improve animal health, productivity, and welfare. As discussed in this review, innovative tools such as the CRISPR/Cas9 system, base editing, and prime editing have established a transformative approach, enabling precise, heritable genetic modifications once deemed unattainable. These technologies have propelled the industry toward an era in which critical traits such as disease resilience, feed efficiency, and reproductive capability can be meticulously optimized, far surpassing the limitations of traditional breeding methods. Gene editing serves as a cornerstone for addressing the primary challenges of swine production, including the need for greater genetic diversity and the constraints posed by selective breeding.

Gene editing has already been proven instrumental in developing disease-resistant pig lines, particularly against pathogens such as PRRSV and ASFV, which cause substantial economic losses worldwide. Furthermore, the technological advances highlighted in this review provide means to enhance complex traits related to growth, fertility, and adaptability, equipping the livestock industry with powerful tools to meet the increasing demands of the growing global population and climate-related challenges. The implications of these advancements are profound because gene editing positions itself as a revolutionary technology with the potential to substantially reduce production costs, improve meat quality, and support sustainable livestock practices. For instance, gene-edited pigs with enhanced disease resistance would not only mitigate economic losses but also align with global goals to decrease antibiotic usage, promoting a safer and more environmentally sustainable approach to livestock management.

However, challenges remain, including the need to mitigate off-target effects, address mosaicism, and achieve efficient gene delivery. While off-target effects are often cited as a challenge in gene editing, they have not proven to be a major issue in livestock applications, particularly with the advancements in newer technologies, such as improved CRISPR systems and precision delivery methods. These developments have significantly minimized unintended genetic changes, making off-target effects less of a concern in the context of livestock genetic editing. Ethical and regulatory considerations, which vary widely across regions, necessitate transparent engagement to build public trust and foster broad acceptance. Integrating gene editing with high-throughput sequencing, big data analytics, and artificial intelligence promises unparalleled precision in genetic selection and management. Achieving these benefits will require cohesive collaboration across research, industry, and regulatory bodies to harmonize standards, promote innovation, and realize the full potential of gene editing as the cornerstone of a resilient, productive, and ethically responsible livestock sector for future generations.

## Figures and Tables

**Table 1:** Detailed overview of sequencing technologies and their applications in swine production.

Generation	Key Technology	Key Features	Advantages	Limitations	Applications in Swine Production	References
First-generation	Maxam-Gilbert sequencing	Chemical cleavage method, radiolabeled DNA, gel electrophoresis	First DNA sequencing method, high precision	Use of hazardous chemicals, labor-intensive, limited scalability	Historical significance in early genetic studies, foundation for sequencing technologies	[124]
	Sanger sequencing	Chain termination using ddNTP, gel electrophoresis, capillary electrophoresis-based	High accuracy, foundational for DNA sequencing	Low throughput, labor-intensive, expensive per base	Initial mapping of QTLs linked to growth, meat, quality, and disease resistance Discovery of major alleles influencing production traits	[22]
Second-generation	Illumina sequencing-by synthesis, Roche 454 pyrosequencing	High-throughput short-read sequencing, parallelization of DNA reads	Reduced cost, scalability, ability to sequence entire genomes	Short read lengths, challenges in resolving complex genomic regions	*Sus Scrofa* 10.2 reference genome assembly, GWAS for traits such as reproduction and feed efficiency, identification of CNVs influencing immune response and metabolic traits, large-scale population re-sequencing	[125,126]
Third-generation	PacBio SMRT, Oxford Nanopore	Long-read sequencing without amplification, direct detection of DNA/RNA molecules	Long reads, detection of epigenetic modification, reduced PCR amplification biases	Higher error rates than second-generation methods, higher cost per read	Identification of structural variants related to fat deposition and meat quality, analysis of alternative splicing events, detection of mobile genetic elements associated with genome stability, characterization of full-length transcripts for more accurate gene expression profiles	[128]
Fourth-generation	Single-cell sequencing, spatial transcriptomics	Single-cell resolution, ability to map gene expression to specific locations within tissues	Cellular heterogeneity analysis, spatial mapping of gene expression	High computational demands, limited by cell capture efficiency	Profiling immune cell populations to study disease resistance, linking tissue-specific gene expression to traits such as muscle development, visualization of spatial gene expression for economically important traits such as fat deposition and skeletal muscle composition	[131]

Abbreviations: CNV, copy number variation; ddNTP, dideoxynucleotides; GWAS, genome-wide association study; PacBio, Pacific Bioscience Inc.; PCR, polymerase chain reaction; QTL, quantitative trait loci; SMRT, single-molecule real-time.

## Data Availability

The original contributions presented in this study are in this article; further inquiries can be directed to the corresponding author.
